# Large-Scale Agile Transformation: A Case Study of Transforming Business, Development and Operations

**DOI:** 10.1007/978-3-030-49392-9_8

**Published:** 2020-05-06

**Authors:** Nils Brede Moe, Marius Mikalsen

**Affiliations:** 6 University of Oslo, Oslo, Norway; 7 Monash University, Clayton, VIC Australia; 8 IT University of Copenhagen, Copenhagen, Denmark; 9 University of British Columbia, Vancouver, BC Canada; SINTEF, Strindvegen 4, 7465 Trondheim, Norway

**Keywords:** Large-scale agile transformation, Agile methods, Large-scale, Case study

## Abstract

Today, product development organizations are adopting agile methods in units outside the software development unit, such as in sales, market, legal, operations working with the customer. This broader adoption of agile methods has been labeled large-scale agile transformation and is considered a particular type of organizational change, originating in the software development units. So far, there is little research-based advice on conducting such transformations. Aiming to contribute towards providing relevant research advice on large-scale agile transformation, we apply a research-based framework for evaluating organizational agility on a product development program in a maritime service provider organization. We found that doing a large-scale agile transformation involves many significant challenges, such as having a shared understanding of the problem, getting access to users, and getting commitment to change that needs to be done. In order to overcome such challenges, we discuss the need for a holistic and integrated approach to agile transformation involving all the units linked to software development.

## Introduction

Software development teams are currently working on developing products providing new digitally enabled customer experiences - while simultaneously incubating and accelerating digital innovations - are facing increasingly complex problems to be solved. Part of the complexity is because solving such problems involves relying on several actors outside of the agile software development team [1, 2]. One example is close cooperation with the business development unit needed in order to achieve the potential advantages of a continuous business and development process [3]. Another example is the need for fast feedback from the customer, which in agile software development is realized by the introduction of frequent software releases to the customer or market. Further, a transformation to continuous delivery needs to consider units such as operations (i.e., the customer-facing side of the organization) and sales and marketing [4]. Agile software teams cooperating with other non-agile units represent a challenge [8], as agile software teams work highly iterative in a sense and respond manner. Other units may be more plan and document-driven. The need for agile software development teams to interact with other units in the organization dynamically and responsively is why companies today aim to scale agile methods beyond software development. We understand such scaling as a large-scale agile transformation in the organization.

As agile methods scale and more units in the organization or entire organizations become agile, it is referred to as organizational agility. Overby et al. [5] define organizational agility as “the ability of firms to sense environmental change and respond appropriately,” and show the different combinations of sensing and response capabilities that organizations should have. They argue that a company that is highly effective at sensing environmental change but is slow to act or acts inappropriately cannot be considered agile. Likewise, a firm that responds appropriately will not be agile if it is unable to sense the correct opportunities to follow. In an agile organization, therefore, if operations sense a change in customer behavior, software development must change the digital customer experience must change, and so must sales and marketing must change accordingly. Worley et al. [6] argue that “agility allows an organization to respond in a more timely, effective, and sustained way than its competitors when changing circumstances require it.” Having the ability to make timely and effective and sustained change results in sustained high performance. Worley et al. (ibid.) introduce a framework to assess organizational agility based on the literature of organization design and flexible and agile organizations. The framework was validated with studies of performance data from 20 firms and interviews with executives. The framework explains routines, the features of these routines, and describes how agile organizations apply them. In order to grasp large-scale agile transformation, we will apply the framework. We chose that particular framework because it is based on organization studies theory and on findings from empirical studies (the framework is detailed in Table 1 in Sect. 2). However, as of yet, few other researchers have tested the framework. Motivated by the need for understanding how agile software development teams can interact with other units, how to do a large-scale agile transformation, and the need for research on frameworks for a large-scale agile transformation we ask the following research question:

### How is a Large-Scale Agile Transformation Done in Practice?

In this paper, we examine large-scale agile transformation in the context of software product development. We understand an agile transformation as broadening the use of agile methods in an organization, that is, involving sales, marketing, development, and operations. The remainder of the paper is organized as follows: In Sect. 2, we present relevant literature on large-scale agile transformation and the agile organization framework we use for understanding such transformation. In Sect. 3, we describe our research method in detail. In Sect. 4, we present results from a case study using the framework. We discuss our findings in Sect. 5. Section 6 concludes and presents key findings from the study.

## Background

In this section, we present existing research on large-scale agile transformation, identify a gap in the research, and suggest a framework for understanding such transformations.

### The Challenges of Large-Scale Agile Transformation

Accelerating rates of technological change, shifting customer behavior, and changing business models and markets necessitate software development that is customer-centric, iterative, continuous, and experimental [1]. Organizations apply agile methods to these digital transformations in order to allow themselves to create, react to, embrace, and learn from change while enhancing customer value [7]. While agile methods have traditionally been practiced within software development teams, there is now a need for using agile methods for interaction between software teams and other non-development organizational units, such as markets, sales, and operations. In practice, this requires a close and continuous linkage between business units (market, sales, and operations) and software development units. The process of continuously assessing and improving this link is described as BizDev [3].

Dikert et al. [8] report that interaction with non-development units using agile methods is the second most challenging aspect of large-scale agile transformations. Challenges include adjusting to an incremental delivery pace, adjusting to product launch activities, and organizational reward models that do not encourage cross-unit collaboration. Working with agile methods across different units, therefore, involves handling an increasing number of actors, interface towards existing systems, and unexpected interdependencies [9]. For organizations with hierarchical and centralized decision-making structures, agile methods cause friction between management that work in traditional ways and agile units [10, 11].

### Transforming Business, Development and Operations

From the above reported practical challenges with a broadening of the agile method towards including business and operation units, there is a need for a theoretical framework that is capable of explaining what is needed to scale agile to the wider organization. To that end, we have chosen to apply a research-based framework for assessing organizational agility [6]. The framework shows that Agile organizations ought to have a set of strategies, structures, and systems that drive them towards higher performance and business agility. Four routines of agility are key:

- Strategizing: How top management teams establish an aspirational purpose, develop a widely shared strategy, and manage the climate and commitment to execution
- Perceiving: The process of broadly, deeply, and continuously monitoring the environment to sense changes and rapidly communicate these perceptions to decision-makers who interpret and formulate appropriate responses.
- Testing: How the organization sets up, runs and learns from experiments.
- Implementing: How the organization maintains its ability and capacity to implement changes, both incremental and discontinuous, as well as its ability to verify the contribution of execution to performance.

The above routines for strategizing, perceiving, testing, and implementing have 14 dimensions, outlined in Table 1 below.

**Table 1.** The conceptual framework for evaluating organizational agility developed by Worley, Williams, and Lawler [6]

Routine	Feature	Description
Strategizing		How top management teams establish an aspirational *purpose*, develop a widely shared *strategy*, and manage the climate and commitment to *execution*
	Sense of shared purpose	The purpose or mission (outcomes other than profit or growth) is widely shared. Values embedded in these statements drive behavior on a daily basis
	Strategic intent	The current business strategy is relevant in today’s market. It clearly distinguishes the firm from other companies and describes the business model (how we make money) but is flexible enough to change on short notice
	Change-friendly identity	There is a clear sense that “who we are” and “what inspires us” aligns with the organization’s brand and reputation. This long-term strategy explains success and encourages the organization to change
Perceiving		The process of broadly, deeply, and continuously monitoring the environment to *sense* changes and rapidly *communicate* these perceptions to decision makers who *interpret* and formulate appropriate responses
	Strong future focus	The organization possesses effective processes for exploring the future deeply
	Maximum surface area structure	The organization is structured in such a way that many people maintain direct and continuous contact with different parts of the business environment
	Vertical information sharing	Information from the environment gets to decision-makers rapidly, in an unfiltered way. Information flows easily, in both directions, between the bottom and top of the organization
	Transparent information	Business, financial, competitor, and organizational information is easily found and widely shared in the organization
Testing		How the organization *sets up, runs, and learns from experiments*
	Flexible resource allocation systems	Capable resources (people, money, time, tools) are available and can be readily deployed to experiment with new ideas
	Encourages innovation	Thinking of new ideas, new businesses, and new ways of working is encouraged in the organization
	Learning capability	Experience with running experiments is captured and applied with each new round so that the company’s capabilities are continuously improved
Implementing		How the organization maintains its *ability and capacity to implement changes*, both incremental and discontinuous, as well as its ability to *verify the contribution of execution* to performance
	Change capability	There is a pragmatic ability to change collective habits, practices, and perspectives. It is embedded in line operations, not isolated in staff groups
	Development orientation	A human resource strategy of building new skills, competencies, and knowledge is clearly articulated
	Flexible reward systems	Incentive systems in the organization—both monetary and nonmonetary—reward both effective performance and change
	Shared leadership	A philosophy that views everyone in the organization as a source of influence and expertise is carried from the top to the bottom

Changing existing organizations is challenging. In some cases, it might be easier to create new adaptable organizations rather than to change an existing organization to be adaptable. However, all organizations have some agile features [12]. An alternative to creating a new organization, therefore, is to start an agile transformation in a part of the existing organizations that already have agile features, which software development units typically do have. The focus in the transformations should be on which features to address to increase agility and how to do it. A part of the organization can, for example, be everyone involved in the development of a product from team management, operation, software development, business, sales, legal, and marketing. In terms of how to do it, as different units are drawn together, it is important to allow for divergent views and opinions to be discussed to allow for transformation to occur. In the concept of “groan zone” [13], it is recognized that everyone has their frame of reference.

Moreover, when people misunderstand one another (which is likely when they all represent different units), they become more confused and impatient. Often, people do not want to be in the groan zone, because it is uncomfortable, but a facilitator can help. The facilitator’s main objective in the Groan Zone is to help the group develop a shared framework of understanding.

## Research Design and Method

In this paper, we report findings from a company that conducted a large-scale agile transformation in one of their product development areas (as suggested above [12]), transforming sales and marketing, software development and operations at the same time. Their product development area is our unit of study and allows us to study how multiple disciplines from multiple organizational units interact when creating a software-based product. Our study is a holistic case study [14]. According to Yin, case studies are the preferred research strategy when a “question is being asked about a contemporary set of events over which the investigator has little or no control” (ibid, p. 9). we followed the five-step process proposed by Yin: 1) Case study design 2) Preparation for data collection. 3) Collecting evidence: execution of data collection on the studied case. 4) Analysis of collected data and 5) Reporting.

We collected data through observations of the collaboration over time in meetings and workshops, through interviews, by studying documentation and by participating in the planning of the agile transformation. The Company (name suppressed for anonymity) is a multinational provider of services the energy, process, and maritime industries, and was chosen because it participated in a research program on large-scale agile software development. The organization had developed a digital solution for booking ship surveys through a web portal. The process in which the digital solution replaced was manually and very costly. Further, booking surveys were sub-optimized, resulting in ships doing surveys in harbors that were not cost-effective and that did not allow all work to be done at once. The potential cost savings from using the digital solution was estimated to be over 10 million Euro per year. The challenge, as we entered the case, was that not cost savings were not sufficient.

### Data Collection and Analysis

Our data collection (Table 2) started in August 2018, when the company needed to rethink the whole product development process in order to reach the estimated earnings of the digital solution. The company recognized that a critical issue was that the missing interaction between software development, sales, marketing, and operations. The missing interaction and the need for improving the product led to a transformation initiative. The researchers participated in all planning meetings of the initiative, lead two of the workshops and had status and synchronization meetings with representatives from the company, and conducted several interviews with key stakeholders. Besides, four large international customers were visited and interviewed. These customer interviews covered the following topics: Describing the customer business process and model, understanding the survey ordering process, reflecting on the usability of the new technology introduced. All activities were documented by taking notes, meeting minutes, and pictures of materials produced in the workshops. Also, we got access to product documentation, contracts, data on user activity on the digital portal, and plans. We ended the data collection in September 2019. The results from the transformation were presented back to the practitioners involved regularly in feedback meetings. More details about the case, the product, and the large-scale agile transformation is found in the results.

**Table 2.** Data sources

Data	Description
Interviews	11 interviews. 1 business developer (2 times), 1 product manager (3 times), 1 development manager, and 1 portfolio manager. Interview with 4 major international customers
Planning meetings, workshops and feedback meetings	2 workshops where conducted with stakeholders from all units. The results of the workshops were analyzed and presented back to the participants in feedback meetings. Additional data was collected from planning meetings
Documents and user behavior statistics	Analyzed user behavior (data gathered from the portal), strategic documents, roadmaps, innovation plans and contract templates

We used a variety of strategies to analyze the material [15]. First, we described the project and context in a narrative to achieve an understanding of what was going on in the large-scale agile transformation project. Then, we described aspects of the transformation by using a framework for assessing organizational agility [6] and analyzing the different routines proposed by the framework (as introduced in Table 1). Further, we analyzed the data by mapping it to the continuous processes described by Fitzgerald et al. [3] (i.e., continuous planning, development, and operations) to understand which processes were disconnected. We hypothesized that the disconnected processes were a core reason for why the company did not realize the potential of the solution. Then we categorized the data according to the organizational agility framework [6]. In the analysis, we emphasized how the need for change was interpreted by different participants in the transformation.

## Results

We first describe how the need for an agile transformation was detected (diagnosing phase), then we describe the outcome of the main activities in the transformation workshops and the work done after each workshop. Based on the results, we identified an understanding of how elements of a large-scale agile transformation are dealt with in practice.

### Diagnosing

**Fig. 1.**
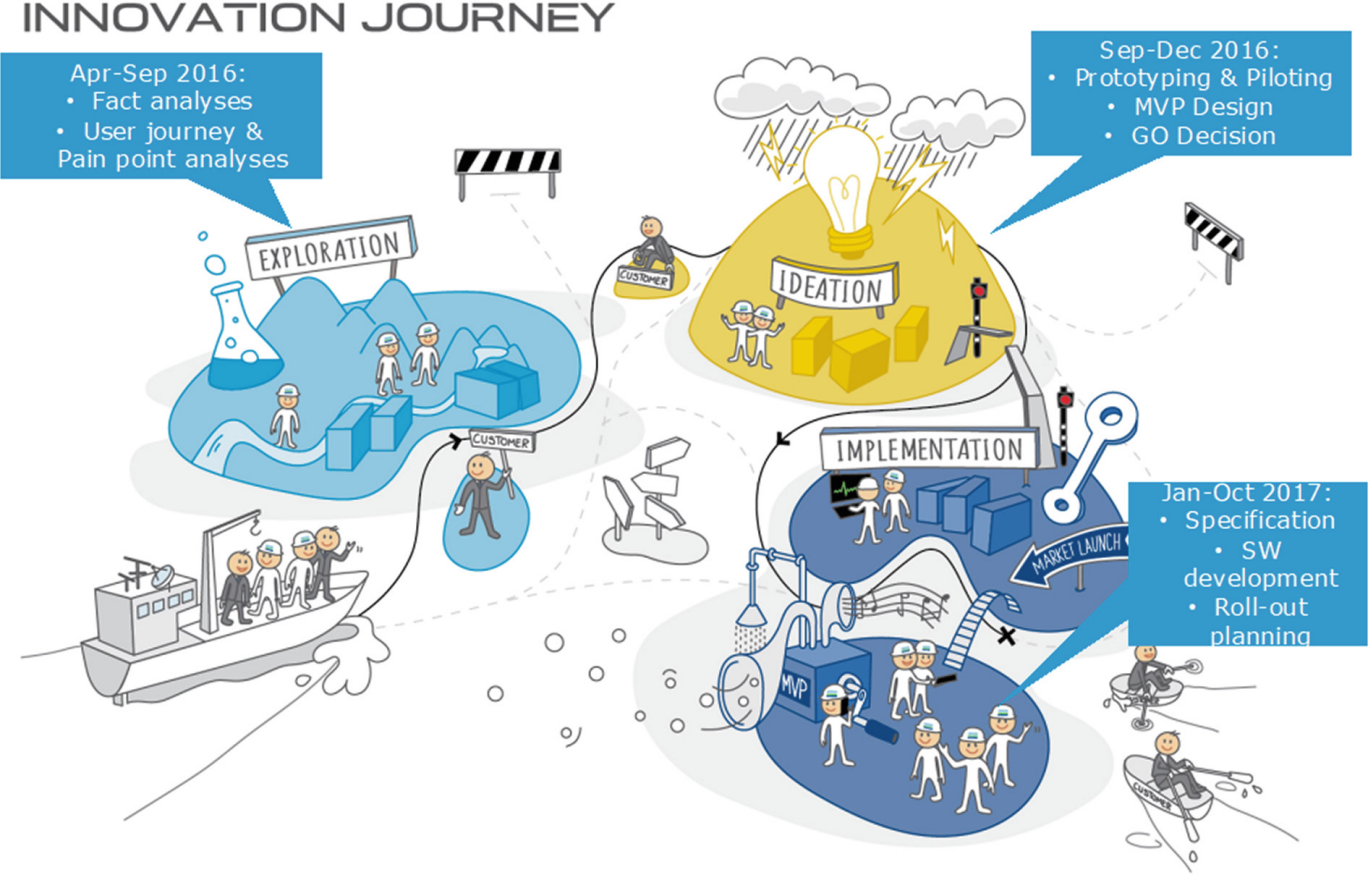
The innovation journey from 2016–2017.

The new product was initiated in 2016, and the goal was to create a system for ship owners to book services for their ships through a portal instead of using the previous manual process. Booking through the portal makes it possible to suggest what combination of services to offer, and when the service should be conducted on a specific ship in a specific port. A system based on machine learning could potentially reduce the cost of surveyor traveling, reduce the total number of services needed, the need to offer services in expensive ports, and reduce the time a vessel needs to be in a port. Both the customers and the company could gain significant savings by replacing the manual booking process. The product development of the booking portal, which also included machine learning, went through several phases, from exploration, ideation to implementation. The work started in April 2016 by an analysis of the market, customer needs, and a concept study. A version of the product was tested in June 2017, and the product was launched in October 2017. Figure 1 shows the innovation journey as described by the company. All managers in the company got training in the innovation method. The planned functionality was implemented and launched, but the product did not meet its expectations. While the software development team implemented all the requested functionality, still only 30% of the customers followed the recommendation by the digital booking process.

Further, the customers, in general, did not accept the recommendations provided by the planning part of the booking system. Recommendations were related to what services a ship should have, in which port the job should be done in, and when the service should happen. Further, there were many customer complaints regarding invoices. It became clear that there was confusion among some customers regarding the service ordered and the service provided by the company. Because most customers did not use the new booking process and, in general, did not accept the recommendations provided by the system, the cost savings were assessed to be very limited in August 2018.

A diagnosing workshop was initiated involving experts from software development and business and customer insight. The diagnosing workshop concluded that the lack of change in user behavior (such as lack of use) could not be explained by the design of the portal and challenges with the user interface alone. The workshop concluded on the following explanations on why the environed results were not achieved:

- The company lacked important information on how the customers actually conducted the former manual booking process.
- The overall company strategy was not aligned with the product strategy. While the new booking system required the company to offer services in limited ports, the company did not achieve a reduction in the number of ports where they had to offer services. Parts of the company still had wanted services to be provided in these ports, even if it was not cost-effective for the company as a whole.
- Internal processes were not coordinated to help the customer in changing his behaviour (e.g., contracts, support, customer contacts).

A transformation was initiated to make all involved units in the company work together to change how they offered the digital service and then change customer behavior. The maritime sector is an old and traditional sector, which makes changes in business processes in the sector slow. Further, this transformation would enable the company to sense the customer needs better, and then respond to the needs as they change. The agile transformation needed to include the following, different organizational units: software development, legal, market, sales, business, and operations. The software development department had been working in an agile way since 2008 and was experienced in using agile, while the other units were still working in a non-agile way. It was agreed to conduct several workshops involving key stakeholders from all the different units. The question was how to conduct workshops to accelerate a transformation that would enable a large part of the company to sense and respond?

Different stakeholders from very different units in the company would necessarily represent different cultures, practices, and ideas. The workshop then needed to facilitate a period of divergent thinking before they could enter the “groan zone”. After a group of diverging ideas brainstormed a list, they found it challenging to discuss the ideas. Everyone had their frame of reference coming from the different units. Moreover, as people misunderstood each other, they become more confused and impatient. The researchers acted as facilitators in the workshops and helped guide the group through the groan zone.

### Unfiltered Access to Customer Insight and Aligning Strategies

The first workshop had representatives from key internal stakeholders, such as customer insight, software development, and data analysis (analyzing the customer data), business, sales, and marketing. The highly cross-functional group had the authority to change the future direction of the technical solution and company internal processes. Each stakeholder was responsible for changes in their unit. The focus shifted from: “how do we provide a better user interface to change customer behavior,” to “what changes do we need to implement in our organization to be able to change customer behavior.” It became evident that to deliver an improved service in fewer ports, the company had to reduce the number of other ports in which the service was delivered. However, then some service stations around the world had to be closed down, and this needed top management support. Such significant changes created internal resistance, as a part of the organization would then need to reduce its service offerings and, as a consequence, would earn less money. Through workshops and meetings, the cross-functional group concluded:

- How certain parts of the sales unit operated where hindering part of the product development organization from meeting directly with the customer, which hindered a more in-depth customer insight. To better understand who uses the new system and those who do not use it, there was a need for direct contact between the software development department and the customers. Several of the previous decisions related to product development were made on wrong assumptions.
- For the company to adjust internally (e.g., stopping offering services in some ports), it was essential that cost and performance are measured on the company level and not per organizational unit. Since costs traditionally were measured per unit, each unit that will reduce their income will resist changes. There was a need to work closely with the world regions that needed to change their offerings of ports. New KPIs (key performance indicators) needed to be set for the whole company, not for individual units.
- Better understand the link between the new business model of the company and how the model is linked to a change in customer behavior. Involving the service planning unit in order to change future contracts was seen as a critical measure.
- Better use of statistics on user behavior in the portal. There is a need to continue analyzing patterns of various customer behavior, and to generate new Power BI Reports.
- Use Machine Learning in a new way to understand better which services to provide in which ports, and which services not to offer.

### Testing, Implementing and New Improvement

Based on more unfiltered access to the customer, new parts of the organization got access to new and essential insights. The situation was further improved by organizing meetings with valuable customers and by insight from interviewing these customers. The interview guide was targeted to understand the enablers of barriers to changing customer behavior. As a result, more insights into customer behavior were generated, particularly on the internal business processes that happened before the customer used the portal i.e., the customers’ internal planning process. Insight was also gained on what was most valuable when the customer made choices in the portal. Through the insight gained through the interview it was found why the customers did not accept recommendations from the new system. The customer behavior was driven by the need of making sure the ship was always operating, and therefore a familiar port is associated with less risk for the customer. One customer commented: “*The port predictions for container vessels are of no benefit because it does not propose ports I prefer.*”

As a result, from the customer interview process, it was concluded that there was a need to understand how the booking system better could support customer preferences, and further insight was needed on how to enable the customer to order services in an unfamiliar port. Knowledge from the workshops and customer activities was fed into the survey planning centers of the company (the planning centers that were spread around the world were engaged in helping customers plan their work). Changes in how the planning centers operated based on new insight is an example of the operational units change the way they deliver the services.

### Next Steps

After changing the software and how the company interacted with the customers, it became evident that the changes had helped to result in the company starting to improve earnings on the product and service they provided radically. However, at the same time, it was clear that the agile transformation now also needed to include more unites, and that it would be an ongoing process with no specific end state.

The company started testing new functionality, which started changing customer behavior. Further, from a more in-depth analysis of the interviews, the need to continue pushing the customers to change their behavior by developing new contracts was considered an essential next step. Involving contract responsibilities in this phase was vital, and the second workshop was conducted. However, it became clear that to get the full effect of new features in the system; there was a need to segregate customers into two segments and to identify the service levels for these segments. Creating customer segments also put forward demands for contracts that would support the segments and, at the same time, needed to enable the customer behavior change to continue. The need for what was known as “smart contracts” was agreed upon in the last workshop. However, what a smart contract looked like was not fully understood.

Further, new questions emerged: is the salesforce ready to sell new products and negotiate new contracts for new customer segments? Are the customers ready to be offered different levels of services based on different contracts (the maritime sector is an old and conservative business)? It became evident in the workshop that sales and legal unites needed to be linked closely with the product development, and that future work was needed in this area.

### Evaluating of Organizational Agility Using the Agility Framework of Worley

The agility framework defined by Worley et al. guided our agile transformation. The framework includes routines for strategizing, perceiving, testing, and implementing and has 14 dimensions (Table 1). To describe how a large-scale agile transformation is done in practice, we then mapped our findings into the framework (Table 3).

**Table 3.** An evaluation of organizational agility using the agility framework of Worley, et al. [6]

Routine	Feature	Results/improvements
Strategizing	Sense of shared purpose	Everyone was aware of the purpose of the program. To make the customer book in the portal and to make smart bookings. However, the units sub-optimized their performance by focusing on their own goals. The need to Involve all units and to align them from the beginning was not understood. It was not until the product was launched and experimented with, and the lack of earnings became clear, that the shared purpose was understood
	Strategic intent	The strategic intent of digitalizing and transforming a traditional sector (maritime) was not unique, however, the combination of applying machine learning, domain knowledge, and customer and vessel data were considered a new business strategy in the market. It became clear that the changes in customer behavior and technology also resulted in the need to change business models (such as sales)
	Change-friendly identity	The new product aligns with the company brands (removed because of the need for anonymity). There is a strong focus on innovation, and all managers have been through management courses. Pressure on cost due to increased competition leads to a continuous search for innovation through digitalization
Perceiving	Strong future focus	The company had a strong focus on digitalizing the maritime industry, and to use the market position and domain insights to do so. Having the willingness to launch such projects and to continue working with the clients on the challenges shows signs that they are working to explore the future by experimenting with new digital solutions and business models
	Maximum surface area structure	While it was evident that the part of the organization was missing direct contact with the customer, this changed throughout the transformation initiative. Throughout the change process, the key to success was unfiltered access to customers from the development side and to increase their ability to sense the need of the customer. Easy access to customers was particularly important since the customer did not always know what they wanted
	Vertical information sharing	It took a long time for the organization to change – over a year. We did not investigate this issue in particular, but the time it took to change can be considered as an indication that there is room for improved vertical information sharing
	Transparent information	The information was not accessible across units in the beginning. However, bringing key stakeholders from different units together in targeted workshops and focusing on collecting and presenting relevant and indicative data helps. The workshop also helped in removing misconceptions (such as reasons for a solution not being used)
Testing	Flexible resource allocation systems	The portal was easy to change, as it did not require much development capacity. However, there was a delay when new requests emerged, as development resources can only be dedicated to fixed periods. They had many other projects to attend. Further, because of people being busy, it took a long time to get all the people from different units to meet
	Encourages innovation	The innovation was partly bottom-up, in that suggestions and ideas could come from everyone. However, resistance emerged when new ideas challenge the existing business models and could disrupt the existing operations of the company
	Learning capability	They had constant feedback from experiments through MVP that could be launched fast to the customer. Further, there was a willingness to experiment with organizational development. Getting people together across units did not require extra funding
Implementing	Change capability	There was a low level of the hierarchy, and easy to get people from different units into the same room to discuss. Throughout the product development habits were changed; however, we did not research to what degree they spread to the next project
	Development orientation	We did not investigate the human resource strategy
	Flexible reward systems	We found that having a monetary reward system based on individual units as limiting the potential of the digital solution. The sales apparatus that was based in certain regions did not have sufficient incentives to push customers over to the digital solution, as this could limit their potential rewards
	Shared leadership	When there was identified a need to work across unites to change the organization and the product, it was not a problem to get access to the needed expertise. Moreover, the expertise had decision making authority, even though senior management needed to be informed

## Discussion

Large-scale agile transformation is a critical issue in responding to the digital transformations that are ongoing in many sectors [1]. Several barriers to such transformation seen from industry experience have been identified, for example, change resistance [8], and inter-team coordination challenges [16]. While conceptual solutions such as continuous development and BizDev has been suggested [3], there is a lack of research-based advice on how agile transformations are to be performed in practice. Driven by our research question – *How is a large*-*scale agile transformation done in practice?* - we have reported findings from a case study of a maritime service provider that aimed to transform service bookings digitally. In the following, we answer this research question by discussing our findings in light of a research-based framework on agile organizations [6].

We found how a typical innovation journey was followed, a product was developed and launched, but the economic gains did not meet expectations. Importantly, as a consequence, the company started investigating the reasons why the product did not meet its projected earnings. One critical insight during the diagnosing phase was that it was not the design of the digital solution per se that caused the lack of customer uptake. For the solution to have its envisioned effects, it would require a change in the internal organization to be able to change customer behavior. The company started a change in a product development environment that already had some agile features, i.e., including software development that was already using agile methods, as suggested by [12]. The change process was done in order to improve its capacity for sensing customer behavior and adapting the digital solutions. The software development and business development units needed more unfiltered access to customer behavior. This is in line with [5], who argue that both sensing and adapting is essential for organizational agility.

We analyzed the steps taken by the unit under study in light of a research-based framework on agile organizations [6]. We found how the part of the organization doing a large-scale organizational transformation addressed all four routines in the framework. The first routine, strategizing, involved struggles to get a sense of shared purpose across the organization, changing business model in terms of offering services in fewer ports, and being committed to change. The second routine, perceiving, involved being willing to experiment with new products, being able to change who gets access to customer insight and that bringing stakeholders together across different units is a critical activity in enabling change. The third routine, testing, we found that it was not changing the technical parts of the system that was the most challenging, but rather to being able to experiment with the organization and making the necessary changes. The fourth routine, implementing, we found that monetary rewards systems and involvement of expertise with decision-making authority were vital in making transformation occur. The challenges and steps taken are in line with Dikert et al. [8] findings that show how integrating non-development units can be restricted to reward models that do not encourage cross-unit collaboration.

Our findings indicate that some of the frictions agile methods can cause [7], such as when the new portal started changing the business model of the sales apparatus. Such frictions indicates that large-scale agile transformation needs new decision structures, which means that a company needs to move from a hierarchical decision structure, and isolated decision structures for each department or unit, to a decision structure across the operational and strategic level of individual units.

Finally, we found that an agile transformation is an ongoing process and that the output of an agile transformation is more continuous processes covering several units, many of which are outside software development. A critical insight is that continuous processes require continues learning and continuous experimentation [3]. Our mapping of findings from the case to the agility framework presented in Table 3 signifies the need for continuity.

### Limitation and Future Research

The main limitations of our study are the single-case design and the possibility of bias in data collection and analysis. The fact that we used a single-case holistic design makes us more vulnerable to bias and eliminates the possibility of direct replication or the analysis of contrasting situations. Therefore, the general criticisms about single-case studies, such as uniqueness and special access to key informants, may also apply to our study. However, our rationale for choosing the company as our case was that it represents a critical case for explaining the challenges of conducting a large-scale agile transformation in practice. Our mode of generalization is analytical, i.e., we used a previously developed framework as a template with which we compared the empirical results of the case study, which is similar to Yin’s [14] concept of Level Two inference.

Another possible limitation is that we based much of our data collection and analysis on semi-structured interviews [17]. The use of multiple data sources made it possible to find evidence for episodes and phenomena from more than one data source; we also observed, talked to, and interviewed the project members over a period of 13 months, which made it possible to study the phenomena from different viewpoints as they emerged and changed.

The results of this study point out several directions for future research. Firstly, our study highlights several challenges that must be met when conducting a large-scale agile transformation. Accordingly, further work should focus on identifying and addressing other problems that may arise when conducting an agile transformation. Secondly, the framework should be used for studying more mature organizations or departments in order to get a better understanding of the main challenges in such transformations. The observed transformation was the first in the company using the framework. When studying the company doing the next transformation on another product, this should be studied since the case then will be more mature, and other issues from using the framework will emerge.

## Conclusion

We have conducted a 13-month study of professionals in a large-scale agile transformation. Our case study of conducting an agile transformation highlights several significant challenges that need to be overcome for a transformation to be successful. This work reports a case study of how a transformation can be done in practice, and also apply a framework for understanding and conducting such an agile transformation. This work is an essential step in its own right since there is much confusion around terms related to agile transformations, similar to early research on the agile transformation of teams [18]. The need for a framework for agile transformation outside of the software development unit is evident when one considers the emergence of phenomena such as Enterprise Agile, Beyond Budgeting, DevOps, Lean Startups, and many other concepts from business agility in general. These are all indicative of the need for a holistic and integrated approach across all the units linked to software development.
